# Supplementary material for: Complexin induces a conformational change at the membrane-proximal C-terminal end of the SNARE complex
Source: eLife. 2016 Jun 2;5:e16886. doi: 10.7554/eLife.16886 (PMC4927292; doi:10.7554/eLife.16886)
Supplement: Figure 2—source data 1. — DOI: http://dx.doi.org/10.7554/eLife.16886.005 [file elife-16886-fig2-data1.docx]

Figure 2 –source data 1

|  | Alexa 647 label site | Alexa 555 label site | dN-SB | % other subconfigurations  (SX:SB) | Number of analyzed traces |
| --- | --- | --- | --- | --- | --- |
| SFC1 | SX 259 | SB 91 | - | 46.3 ± 3.1 | 87 |
| SFC2 | SX 249 | SB 82 | - | 47.4 ± 4.2 | 103 |
| SFC3 | SX 193 | SB 28 | - | 47.2 ± 1.0 | 110 |
| SFC1 | SX 259 | SB 91 | + | 13.5 ± 1.4 | 108 |
| SFC2 | SX 249 | SB 82 | + | 3.1 ± 4.3 | 91 |
| SFC3 | SX 193 | SB 28 | + | 9.22 ± 2.6 | 100 |
