# Supplementary material for: Complexin induces a conformational change at the membrane-proximal C-terminal end of the SNARE complex
Source: eLife. 2016 Jun 2;5:e16886. doi: 10.7554/eLife.16886 (PMC4927292; doi:10.7554/eLife.16886)
Supplement: Figure 3—source data 1. — DOI: http://dx.doi.org/10.7554/eLife.16886.007 [file elife-16886-fig3-data1.docx]

Figure 3–source data 1

|  | Alexa 647 label site | Alexa 555 label site | % *cis* conformation | Number of analyzed traces |
| --- | --- | --- | --- | --- |
| SX-S25-SB-Cpx WT | SX 249 | Cpx 24 | 62.4 ± 2.7 | 86 |
| SX-S25-SB-Cpx SC | SX 249 | Cpx 24 | 71.8 ± 3.3 | 56 |
| SX-S25-SB-Cpx NC | SX 249 | Cpx 24 | 28.5 ± 2.3 | 27 |
