# Supplementary material for: Complexin induces a conformational change at the membrane-proximal C-terminal end of the SNARE complex
Source: eLife. 2016 Jun 2;5:e16886. doi: 10.7554/eLife.16886 (PMC4927292; doi:10.7554/eLife.16886)
Supplement: Figure 4—source data 1. — DOI: http://dx.doi.org/10.7554/eLife.16886.010 [file elife-16886-fig4-data1.docx]

Figure 4–source data 1

|  | Alexa 647  label site | Alexa 555 label site | Conditions | % conformational change | Number of analyzed traces |
| --- | --- | --- | --- | --- | --- |
| SFC1 | SX 259 | SB 91 | 1.0 μM Cpx WT [1-134] | 42.1 ± 4.2 | 120 |
| SFC2 | SX 249 | SB 82 | 1.0 μM Cpx WT [1-134] | 35.5 ± 6.1 | 103 |
| SFC3 | SX 193 | SB 28 | 1.0 μM Cpx WT [1-134] | 4.3 ± 0.2 | 112 |
| SFC1 | SX 259 | SB 91 | 1.0 μM Cpx WT [26-134] | 10.7 ± 6.4 | 51 |
| SFC2 | SX 249 | SB 82 | 1.0 μM Cpx WT [26-134] | 9.7 ± 3.9 | 67 |
| SFC1 | SX 259 | SB 91 | 1.0 μM Cpx WT [48-134] | 11.2 ± 4.9 | 60 |
| SFC2 | SX 249 | SB 82 | 1.0 μM Cpx WT [48-134] | 11.6 ± 6.2 | 30 |
| SFC2 | SX 249 | SB 82 | no Cpx | 6.8 ± 1.4 | 92 |
| SFC2 | SX 249 | SB 82 | 1.0 μM Cpx WT [1-134] | 35.5 ± 6.1 | 103 |
| SFC2 | SX 249 | SB 82 | 1.0 μM Cpx SC [1-134] | 41.9 ± 2.1 | 70 |
| SFC2 | SX 249 | SB 82 | 1.0 μM Cpx NC [1-134] | 9.9 ± 0.2 | 77 |
| SFC2 | SX 249 | SB 82 | 1.0 μM Cpx 4M [1-134] | 5.5 ± 0.6 | 85 |
| SFC2 | SX 249 | SB 82 | no Cpx | 6.8 ± 1.4 | 92 |
| SFC2 | SX 249 | SB 82 | 0.001 μM Cpx WT [1-134] | 11.5 ± 2.1 | 96 |
| SFC2 | SX 249 | SB 82 | 0.1 μM Cpx WT [1-134] | 23.6 ± 3.2 | 95 |
| SFC2 | SX 249 | SB 82 | 1.0 μM Cpx WT [1-134] | 35.5 ± 6.1 | 103 |
| SFC2 | SX 249 | SB 82 | 10.0 μM Cpx WT [1-134] | 44.7 ± 6.8 | 107 |
