# Supplementary material for: Complexin induces a conformational change at the membrane-proximal C-terminal end of the SNARE complex
Source: eLife. 2016 Jun 2;5:e16886. doi: 10.7554/eLife.16886 (PMC4927292; doi:10.7554/eLife.16886)
Supplement: Table 2—source data 1. — DOI: http://dx.doi.org/10.7554/eLife.16886.013 [file elife-16886-table2-data1.docx]

Table 2–source data 1

|  | γ (no Cpx) | γ (Cpx) |
| --- | --- | --- |
| SFC1 | 1.94 | 1.83 |
| SFC2 | 1.61 | 1.46 |
