# Supplementary material for: Complexin induces a conformational change at the membrane-proximal C-terminal end of the SNARE complex
Source: eLife. 2016 Jun 2;5:e16886. doi: 10.7554/eLife.16886 (PMC4927292; doi:10.7554/eLife.16886)
Supplement: Figure 6—source data 1. — DOI: http://dx.doi.org/10.7554/eLife.16886.017 [file elife-16886-fig6-data1.docx]

Figure 6–source data 1

| Alexa 647 labeled  protein complex | Alexa 647 label site | Alexa 555 labeled  protein complex | Alexa 555 label site | k_off_ (s^-1^) | k_on_ (μM^-1^s^-1^) | Apparent K_D_ (μM) | Number of analyzed traces | Number of transitions |
| --- | --- | --- | --- | --- | --- | --- | --- | --- |
| SX-S25-SB-Cpx WT | Cpx 24 | SX-S25 | SX 249 | 2.16 ± 0.02 | 0.34 ± 0.07 | 6.46 ± 1.2 | 109 | 204 |
| SX-S25-SB-Cpx SC | Cpx 24 | SX-S25 | SX 249 | 1.36 ± 0.06 | 0.56 ± 0.06 | 2.42 ± 0.3 | 125 | 265 |
| SX-S25-SB-Cpx NC | Cpx 24 | SX-S25 | SX 249 | 2.37 ± 0.2 | N.A. | N.A. | 36 | 7 |
| SX-S25-SB-Cpx 4M | Cpx 24 | SX-S25 | SX 249 | N.A. | N.A. | N.A. | 3 | 0 |
