# Supplementary material for: Complexin induces a conformational change at the membrane-proximal C-terminal end of the SNARE complex
Source: eLife. 2016 Jun 2;5:e16886. doi: 10.7554/eLife.16886 (PMC4927292; doi:10.7554/eLife.16886)
Supplement: Figure 8—source data 1. — DOI: http://dx.doi.org/10.7554/eLife.16886.020 [file elife-16886-fig8-data1.docx]

Figure 8–source data 1

| Alexa 647 labeled protein/  protein complex | Alexa 647 label site | Alexa 555 labeled protein | Alexa 555  label site | k_off_ (s^-1^) | k_on_ (μM^-1^s^-1^) | Apparent K_D_ (μM) | Number of analyzed traces | Number of transitions |
| --- | --- | --- | --- | --- | --- | --- | --- | --- |
| SX | SX 249 | Cpx WT | Cpx 26 | 2.96 ± 0.44 | 0.16 ± 0.001 | 18.5 ± 2.8 | 75 | 54 |
| SX-S25 | SX 249 | Cpx WT | Cpx 26 | 1.8 ± 0.07 | 0.92 ± 0.03 | 2.0 ± 0.03 | 99 | 406 |
| SX-S25-SB | SX 249 | Cpx WT | Cpx 26 | 0.12 ± 0.03 | 1.62 ± 0.1 | 0.074 ± 0.01 | 183 | 1104 |
| SX-S25-SB | SX 249 | Cpx SC | Cpx 26 | 0.12 ± 0.02 | 1.89 ± 0.1 | 0.063 ± 0.01 | 214 | 1652 |
| SX-S25-SB | SX 249 | Cpx NC | Cpx 26 | 0.12 ± 0.01 | 1.75 ± 0.1 | 0.068 ± 0.01 | 258 | 2568 |
| SX-S25-SB | SX 249 | Cpx 4M | Cpx 26 | 1.34 ± 0.01 | N.A. | N.A. | 40 | 5 |
